# Impact of Twice-weekly Scheduled Dialysis Through the Emergency Department for Patients with End-stage Renal Disease

**DOI:** 10.5811/westjem.31053

**Published:** 2025-07-08

**Authors:** Shilpa Raju, Micah Ownbey, Jennifer Cotton, Jamal Jones, Jo Abraham, Christy Hopkins, Emad Awad

**Affiliations:** * University of Utah Health, Department of Emergency Medicine, Salt Lake City, Utah; † University of Utah Health, Department of Internal Medicine, Division of Nephrology and Hypertension, Salt Lake City, Utah

## Abstract

**Introduction:**

Patients with end-stage renal disease (ESRD) who do not have access to standard dialysis often rely on emergency-only dialysis (EOD) through the emergency department (ED). Compared to standard dialysis, EOD leads to higher hospitalization rates, hospital days, and higher mortality. Our objective in this this study was to examine hospitalization rates and total hospital days after transitioning patients with ESRD from ED EOD to scheduled ED dialysis, and subsequently to standard outpatient dialysis.

**Methods:**

We performed this retrospective study at a single, academic teaching hospital over the course of 10 years (2014–2023). Patients >18 years of age who received dialysis primarily through the ED for more than one year were included in the study. We studied two cohorts. Cohort 1 consisted of patients with ESRD who transitioned from ED EOD to twice-weekly ED dialysis. Cohort 2 was composed of patients who were transitioned from twice-weekly ED dialysis to standard outpatient dialysis. We performed paired patient analysis using the Wilcoxon signed-rank test. Primary outcomes included hospitalizations per month and total hospital days.

**Results:**

Overall, there were seven patients in cohort 1 (mean age 39 years, 86% female) and 20 patients in cohort 2 (mean age 44, 50% female). Patients who transitioned to twice-weekly ED dialysis from ED EOD had lower hospitalizations per month (1.44 vs 0.26, *P* <.05) and fewer total hospital days per month (2.18 vs 1.20, *P* < .05). Patients who transitioned from twice-weekly scheduled ED dialysis to standard outpatient dialysis had even lower hospitalizations per month (0.10 vs 0.02, *P* < .01) and total hospital days (0.31 vs 0.08, *P* < .01).

**Conclusion:**

Introducing scheduled twice-weekly ED dialysis sessions for unfunded patients with end-stage renal disease was associated with lower overall hospitalization rates and hospital days than emergency-only dialysis. These measures were decreased further after transitioning patients from ED scheduled dialysis to standard dialysis.

## INTRODUCTION

Undocumented immigrant patients represent a vulnerable population, especially those who carry the burden of chronic illness. It is estimated that there are between 6,000–9,000 undocumented immigrants with end-stage kidney disease (ESRD) in the United States.^1^ In 2019, it was estimated that only 12 states provided statewide access to standard outpatient dialysis (three times a week) for undocumented patients through Medicaid or emergency Medicaid. The remainder of states did not have uniform access to standard dialysis, leaving many patients dependent upon emergency-only dialysis (EOD).^2^–^6^ An estimated 30–50% of undocumented immigrants with ESRD receive EOD, which is provided to patients who present to the emergency department (ED) with critical conditions including life-threatening hyperkalemia, hypoxemia, uremia, and metabolic acidosis.^7^

Although most undocumented immigrants with ESRD are reported to be younger and healthier than US citizens who have ESRD,^8^–^9^ undocumented patients who rely on EOD have been shown to have higher mortality and hospitalization rates, and higher healthcare utilization compared to patients receiving standard outpatient dialysis.^10^–^11^ This has significant implications for EDs that strive to care for this population including increased ED visits, long ED length of stays (LOS) and increased frequency of observation hours.^12^

Transitioning undocumented patients from EOD to standard dialysis has been shown to result in an overall decrease in ED visits, hospitalizations, and blood transfusions.^13^ Furthermore, providing standard dialysis to such patients has been associated with a more than four-fold decrease in average monthly emergency Medicaid service dialysis expenditures.^2^ A study in Texas also reported a net savings of nearly $6,000 per person per month after transitioning patients to traditional dialysis.^11^

The social and emotional impacts of EOD on both patients and clinicians are profound. Undocumented patients often report psychosocial distress due to unpredictable access, episodes of life-threatening illness, distressing symptoms, and the family and social impacts of EOD.^14^–^15^ Undocumented patients who transitioned from emergency to standard outpatient dialysis in Colorado reported improvements in quality of life and symptom burden, and felt that their humanity had been restored.^15^ Clinicians who provide EOD care report emotional exhaustion from witnessing needless suffering and high mortality as well as moral distress from the perception of propagating injustice.^17^–^18^

Our objective in this study was to evaluate hospitalization rates and total hospital days for undocumented patients with ESRD who presented to the ED for EOD and were transitioned to twice-weekly scheduled ED dialysis. Additionally, we examined the same outcomes for patients who were subsequently transitioned from twice-weekly scheduled ED dialysis to standard outpatient dialysis after a Centers for Medicare & Medicaid (CMS) rule change went into effect. We hypothesized that providing twice-weekly ED dialysis would reduce hospitalization rates and shorten the number of total hospital days among undocumented patients with ESRD compared to the period when they received EOD. Additionally, we hypothesized that transitioning to standard outpatient dialysis would result in a further reduction of hospitalization rates and hospital days compared to the twice-weekly ED dialysis.

**Population Health Research Capsule**

What do we already know about this issue?

*End-stage renal disease (ESRD) in undocumented immigrant patients who rely on emergency-only dialysis (EOD) face major health challenges*.

What was the research question?

*We evaluated hospitalization rates and days for undocumented immigrant patients with ESRD transitioning from EOD to scheduled dialysis through the emergency department (ED)*.

What was the major finding of the study?

*When compared to EOD, scheduled ED dialysis reduced hospitalization rates by 1.18 hospitalizations per month (1.4 vs 0.3, P < .05)*.

How does this improve population health?

*Implementation of scheduled ED dialysis for undocumented immigrant patients with ESRD decreased inpatient healthcare utilization*.

## METHODS

### Design and Setting

This study was a retrospective analysis of data collected from patient encounters at an academic ED with an annual volume of 57,000 visits. We obtained data through chart review for records between January 1, 2014–December 31, 2023. Patients were identified by a primary ED diagnosis of ESRD in the electronic medical record. Immigration status was confirmed for each patient via case management records. We included in the study patients who had primarily accessed dialysis care through the ED for at least 12 consecutive months during two consecutive treatment periods (ED EOD and scheduled ED dialysis, scheduled ED dialysis and outpatient dialysis, or all three treatment periods). Patients were excluded from the analysis if they used the ED for dialysis care for <12 consecutive months (ie, the ED was used as a temporary bridge to outpatient dialysis) or if their care only spanned one treatment period (no comparison period for paired analysis).

We identified a total of 109 patients. Of these, 54 patients used the ED on consecutive months during the study period for their dialysis care. Twenty-seven patients required short-term ED dialysis (<12 months). Of these, 11 used the ED during only one treatment period (no comparison period for paired analysis). Median time of ED dialysis for patients in the short-term dialysis group was 4.5 months. Twenty-seven patients required long-term ED dialysis (>12 months); three patients were excluded because they used the ED during only one treatment period (no comparison period for paired analysis). The median time of ED dialysis for the patients who relied on the ED for long-term dialysis care was 29 months.

Data abstraction included total time (in months) that the patient used the ED for dialysis care. Period times were delineated by time (in months) that the patient received emergency-only dialysis (Period 1) in the ED, scheduled ED dialysis (Period 2), and standard outpatient dialysis (Period 3). Scheduled ED dialysis was defined as the time the patient was switched to scheduled twice-weekly dialysis days in the ED until the time the patient was accepted into an outpatient dialysis center. These events were recorded in both case management and nephrology notes. Additional variables included hospitalizations per month and hospital days per month for each of the three treatment periods. A hospitalization was recorded if the patient was admitted to an inpatient service. All hospitalizations were counted whether it was primarily for a dialysis-related issue or for other medical or surgical issues. Lastly, mortality was recorded if the patient death occurred during the study dates. Chart abstraction was performed by one person who was trained prior to chart review. The abstractor was aware of the study hypothesis. The study received an ethical exemption from the institutional review board.

### Dialysis Protocol

Period 1: Prior to August 2016, undocumented patients received EOD through the ED based on the following criteria: hypoxia; hyperkalemia; uremia; metabolic acidosis; or electrocardiogram changes. Patients would present to the ED when they felt that they needed dialysis. After evaluation by an emergency physician, the patient was admitted to the ED observation (OBS) unit (11-bed unit) for dialysis, unless the patient’s condition required inpatient care. The patients would be taken to the inpatient dialysis unit (5-bed unit) for dialysis and then returned to the ED OBS unit after dialysis. During Period 1, patients received two dialysis sessions of 4 hours each within 24 hours to minimize the risk of disequilibrium. Patients with hemoglobin levels <7 grams per deciliter (g/dL) received blood transfusions, but no advanced treatments such as erythropoietin were administered. Patients receiving dialysis through either the ED or ED OBS unit were counted as ED visits.

Period 2: In August 2016, patients using the ED for EOD were instructed to present to the ED on assigned days of the week, instead of when they felt that they needed dialysis. This was an operational decision to help streamline care, reduce ED crowding, and minimize delays in inpatient dialysis caused by the simultaneous presentation of multiple dialysis patients needing EOD on the same day. The nephrology team assigned each patient designated days for dialysis (eg, Monday/Thursday, Tuesday/Saturday). Patients would present to the ED on their designated days and be evaluated in triage by an emergency clinician. After evaluation, stable patients would wait in the ED waiting room until the inpatient dialysis unit was able to accommodate them. The patient would complete one dialysis session and then be discharged by the ED team. Patients were only roomed in the ED if they required temporizing measures or had a condition that required a private room for dialysis (eg, COVID-19). During Period 2, patients would receive dialysis regardless of whether they met criteria for emergency-only dialysis or not.

Period 3: In 2020, the CMS instituted a rule change that allowed undocumented patients with ESRD to receive standard dialysis at outpatient dialysis centers. Once enrolled, patients were assigned to outpatient dialysis centers to receive standard dialysis care and no longer came to the ED for routine dialysis care.

### Program Support

Undocumented patients presenting to the ED for EOD were managed with existing ED resources throughout all three study periods. There were no facility modifications made to either the ED or inpatient dialysis unit during the study. During Period 1, the inpatient dialysis unit added an additional 2.0 full-time equivalents of dialysis nursing staff to accommodate the overall increased dialysis volumes in patients presenting to the ED for EOD. Subsequent dialysis staffing was not adjusted further during other study periods. Inpatient dialysis nurses dialyze a maximum of five patients at a time during a four-hour dialysis session. The estimated nursing cost for eight hours of dialysis per week for one year is $22,058 for a maximum of five patients. Each dialysis session was four hours in duration with an average cost of $2,650 per session. Patients presenting through the ED for dialysis in this study typically required a minimum of two dialysis sessions per week, or eight hours of dialysis per week.

In Period 2, patients who used the ED for EOD were instructed to present to the ED on specific days of the week for their dialysis. This was purely an operational change that allowed stable patients to be assessed in triage and, if stable, wait in the waiting room until the inpatient dialysis unit was ready for them. In contrast to Period 1, where the patient often required an overnight observation stay for their two dialysis sessions, in Period 2 the patient would go home after dialysis and return later in the week for their second dialysis session. The base dialysis sessions/week were similar between Periods 1 and 2. During Period 1, the patients received two dialysis sessions over two consecutive days, and during period 2, the patients had one dialysis session twice a week on assigned weekdays (unless admitted). To our knowledge, the switch to scheduled ED dialysis did not require any additional resources from either the ED or the institution.

In Period 3, after the CMS rule change, undocumented patients who had been using the ED for dialysis became eligible to receive dialysis in an outpatient dialysis center; as a result, the overall ED utilization by this population for dialysis decreased significantly. The overall ED dialysis patient census varied over the 10-year study period and was dependent upon new patients entering the system, funding availability, patient relocation, and mortality. At current state, with the ability to obtain funding for undocumented patients with ESRD for outpatient dialysis, the ED typically manages between three and five patients who are either awaiting funding approval, fistula placement, or fistula maturation.

### Dialysis Patient Management

Dialysis patient management is a collaborative effort between case management, ED leadership, and the inpatient nephrology services. Patients seeking care for dialysis are flagged by hospital case managers who meet individually with the patients to assist them in obtaining funding, when feasible. Initial financial screening typically takes between 1–2 hours per patient. If the patient does not qualify for funding, nephrology and ED leadership are alerted, and the patient is directed to use the ED for subsequent dialysis needs. Patients who do not qualify for funding have no other ongoing interaction with case management. Individual care plans for each patient are developed by ED and nephrology leadership. The care plans are entered and updated in the individual patient record by the ED medical director. When the medical record is accessed, a care management flag alerts healthcare personnel that the patient has a specific care pathway in place.

After the CMS rule change, patients eligible for funding would meet with case management and then would be referred to an outpatient dialysis program. The referral process would typically take two hours per patient. After moving the bulk of these patients over to outpatient facilities, the ED typically manages between 3–5 dialysis patients on an ongoing basis who are either awaiting funding approval, arteriovenous fistula (AVF) placement, or AVF maturation. Currently, the case managers’ time commitment varies between 0–4 hours per week depending on how many new patients are being managed. No additional case manager resources were added during the study period.

### Study Population

The study included undocumented patients, ≥18 years of age who previously received emergency dialysis primarily through the ED for a period of one year or more. We collected data from two cohorts. Cohort 1 consisted of undocumented individuals who initially were receiving ED EOD dialysis and later transitioned to twice-weekly dialysis through the ED. Cohort 2 was composed of undocumented patients who transitioned from twice-weekly ED dialysis to standard outpatient dialysis. Four of the 27 patients studied were represented in both cohorts. This was a paired analysis; thus, patients who only received dialysis during one period (no comparison time frame) were not included in the analysis.

### Variables and Measures

The primary outcomes measured were hospitalization rate and number of hospital days. The hospitalization rate was calculated as the number of hospitalizations per month, and hospitalization days were reported as the total hospital days per month.

### Data Analysis

We summarized descriptive statistics for the baseline characteristics in cohort 1 and cohort 2. Median hospitalization rates and hospital days were calculated for three treatment periods: EOD; twice-weekly ED dialysis; and standard outpatient dialysis. Given that the data were not normally distributed, the Wilcoxon signed-rank test was used to examine the statistical significance and magnitude of the differences in median hospitalization rates and hospital days between patients who transitioned to twice-weekly dialysis from EOD, as well as between patients who transitioned to standard dialysis from twice-weekly dialysis. The Wilcoxon signed-rank test examines the difference between matched pairs for non-parametric data. For the purposes of this study, we compared the primary outcomes for individual patients during each of the different periods. All analyses were performed using SPSS Statistics v29 (IBM Corp, Armonk, NY).

## RESULTS

### Baseline Characteristics

In cohort 1, the study population included a total of seven undocumented patients with ESRD, six (85.1%) of whom were females. The median age for this group was 39 years (interquartile range [IQR] 21–49). Four of the seven patients (57%) had an AVF in place. In cohort 2, there were 20 patients, 10 of whom were females (50%). The group’s median age was 46 years (IQR 32–56). Eight of the 20 patients in cohort 2 had an AVF in place (40%). Table 1 presents the median hospitalizations per month and hospital days for both cohorts by treatment regimen (EOD, twice-weekly dialysis, and standard dialysis).

### Comparison Between Treatment Regimens

Our analysis demonstrated that in cohort 1, transitioning from EOD dialysis to twice-weekly dialysis significantly reduced the median hospitalization rates by 1.18 hospitalizations per month (1.44 vs 0.26, *P* < .05). Additionally, the switch led to a median one-day reduction in total hospital days per month (2.18 vs 1.20, *P* <.05). In cohort 2, our findings revealed that transitioning from twice-weekly dialysis to standard dialysis resulted in significantly fewer hospitalizations per month (0.10 vs 0.02, *P* < .01) and decreased hospital days by a median of 0.23 days per month (from 0.31 to 0.08, *P* < .01) (Table 2).

### Hospitalizations

Inpatient hospitalizations during each period are detailed in Table 3. The percentage of hospitalizations for dialysis-related conditions decreased from 98% in Period 1 to 74% in Period 2, and further decreased to 62% in Period 3. Hospitalizations categorized as directly related to ESRD were inpatient admissions due to hyperkalemia, volume overload, metabolic acidosis, uremia, and/or a combination of the above. Dialysis and non-dialysis-related medical, and surgical admissions are detailed in Table 4.

## DISCUSSION

This study examined the rates of hospitalization and number of hospital days per month for individual patients during two different treatment periods, thus comparing rates for the same patient under different treatment scenarios. Two cohorts were studied. Cohort 1, comprising seven undocumented patients, demonstrated that transitioning from ED EOD dialysis to twice-weekly ED dialysis significantly reduced hospitalization rates by 1.18 hospitalizations per month. Additionally, the switch led to a one-day reduction in hospital days per month. Cohort 2 consisted of 20 patients and demonstrated that switching from twice-weekly dialysis to standard dialysis resulted in significantly fewer hospitalizations per month and fewer hospital days per month compared to the twice-weekly dialysis regimen. Overall hospital admissions for acute management of dialysis-related conditions decreased over the study period.

Our study supports prior reports that have shown that undocumented patients with ESRD treated with EOD have more hospitalizations and spend more days in the hospital than those receiving standard outpatient dialysis.^10^,^11^,^13^ The overall mortality rate for patients in this study was 25% over the 10-year study period. The study did span the peak of the COVID-19 pandemic. The impact of the pandemic on overall mortality of the group is unknown.

To date, no studies have assessed the impact of undocumented patients with ESRD presenting to the ED on scheduled days for dialysis instead of relying on EOD. In this study, scheduled patients with no other acute complaints had a venous blood gas obtained on Day 1 to assess potassium and hemoglobin levels. Blood transfusions were administered for hemoglobin levels <7 g/dL. No additional labs or diagnostic testing were otherwise obtained unless dictated by patient condition or clinician concern. On Day 2 of scheduled dialysis, diagnostic testing was not obtained, unless dictated by patient condition or clinician concern. Dialysis was provided twice weekly regardless of whether the patient met criteria for emergency dialysis. None of the patients in our study received peritoneal dialysis. Our study did show significant reduction in hospitalization and overall number of hospital days when patients were switched to scheduled dialysis but, importantly, our study suggests that better access to healthcare and standard outpatient dialysis is optimal for patient health outcomes and resource utilization.

The study population was limited due to being conducted at a single site with a relatively low estimated number of eligible participants in the study area. It is estimated that approximately 95,000 undocumented residents reside in the state where the study took place.^19^ Based on limited data, the estimated race-adjusted incidence rate of ESRD is nearly 500 cases per 1,000,000. This suggests that there are likely fewer than 50 undocumented immigrants with ESRD in the entire state.^5^ Given our small sample size and skewed data, we employed the Wilcoxon signed-rank test to compare different dialysis protocols. A notable strength of this test lies in its nonparametric nature, which ensures robustness even when dealing with skewed distributions like ours. By allowing us to compare paired observations within the same individuals, the test effectively assessed changes in hospitalization rates and hospital days over time.

## LIMITATIONS

This study has several important limitations that should be noted. Its retrospective design may have introduced biases and limited the ability to establish causality. With respect to adherence to methodological standards in medical record review,^20^ our study had a single abstractor who was not blinded to the study hypothesis, which could have introduced bias into how the data were abstracted. Our study was limited only to patients who used the ED for dialysis for more than a 12-month period, which limits the interpretation of this data for patients who use ED-only dialysis as a short-term bridge to outpatient dialysis. In addition, our study was conducted at a single hospital in one state. Thus, the findings may not be generalizable to other healthcare systems or populations. As well, the relatively small sample size may have reduced the study’s statistical power and external validity. Additionally, financial impacts were not assessed as part of this study. Lastly, we had limited data on potential confounders such as comorbidities, socioeconomic status, and access to healthcare. These uncontrolled factors could have influenced the observed outcomes. Future research should include a larger sample size, multiple healthcare settings, and account for potential confounders to enhance the validity and generalizability of the findings.

## CONCLUSION

In this small, single-site retrospective study, the implementation of scheduled twice-weekly dialysis among undocumented patients with end-stage renal disease through the ED significantly reduced overall hospitalization rate and number of hospital days. Furthermore, transitioning to standard outpatient dialysis resulted in even greater reductions in hospitalizations and hospital days. Further research in this area, with a larger sample size and consideration of other potential confounding factors, would yield valuable insights into healthcare and resource utilization.

## Figures and Tables

**Table 1:** Baseline characteristics in a study of patients with sporadic emergency dialysis in the emergency department (ED) transitioning to twice weekly scheduled ED dialysis.

Cohort 1 (N= 7)		Cohort 2 (N=20)	
	
Age		Age	
Mean ± SD	39.2 ± 15.4	Mean ± SD	44.1 ± 13.6
Median (IQR)	39.0 (21–49)	Median (IQR)	46.0 (32–56)
Sex		Gender	
Male	1 (14.3%)	Male	10 (50.0%)
Female	6 (85.7%)	Female	10 (50.0%)
Emergency-only dialysis		Twice-weekly dialysis	
Hospitalization/month Median (IQR)	1.44 (1.11–1.66)	Hospitalization/month Median (IQR)	0.10 (0.06–0.19)
Total hospital days Median (IQR)	2.18 (1.50–2.55)	Total hospital days Median (IQR)	0.31 (0.18–0.91)
Total hospital days Number of days	15.30	Total hospital days Number of days	13.53
Twice-weekly dialysis		Standard dialysis	
Hospitalization/month Median (IQR)	0.26 (0.12–0.30)	Hospitalization/month Median (IQR)	0.02 (0.0–0.05)
Total hospital days Median (IQR)	1.20 (0.36–2.10)	Total hospital days Median (IQR)	0.08 (0.0–0.33)
Total hospital days Number of days	8.18	Total hospital days Number of days	4.81

*IQR*, interquartile range; *EOD*, emergency-only dialysis.

**Table 2:** Comparison between treatment regimes in a study of patients with sporadic emergency dialysis in the emergency department (ED) transitioning to twice weekly scheduled ED dialysis: Wilcoxon signed-rank test.

Dialysis protocol	EOD	Twice weekly	Difference	*P-*value
Hospitalization/month	1.44	0.26	1.18	.02
Hospital days	2.18	1.20	0.98	.04
Dialysis protocol	Twice weekly	Standard	Difference	*P-*value
Hospitalization/month	0.10	0.02	0.8	.002
Hospital days	0.31	0.08	0.23	.005

*EOD*, emergency-only dialysis.

**Table 3:** Hospitalization by time period.

Time Period	Total Admissions	ESRD	Medical Conditions	Surgical Conditions	*Subtotal*
Dialysis-related Conditions					
Period 1	241	231	4	2	237 (98%)
Period 2	112	66	9	8	83 (74%)
Period 3	47	6	3	20	29 (62%)
Non-dialysis-related Conditions					
Period 1	241		4	0	4 (2%)
Period 2	112		23	6	29 (26%)
Period 3	47		15	3	18 (38%)

*ESRD*, end-stage renal disease.

**Table 4:** Dialysis- and non-dialysis-related hospital conditions.

	Medical issues	Surgical issues
Dialysis-related conditions		
	Uremic pericarditis/pericardial effusion	AV fistula complications
	Bacteremia/endocarditis from infected tunneled dialysis catheter (TDC)	TDC complications requiring surgical intervention
Non-dialysis-related conditions		
	Severe infections	Ischemic gut
	Pneumonia	Amputation due to osteomyelitis
	Sepsis/septic shock	Orthopedic procedures
	Cardiovascular conditions	Parathyroidectomy
	Arrhythmia	Appendicitis
	Cardiac arrest	
	Acute coronary syndrome	
	Neurological conditions	
	Intracerebral hemorrhage	
	Subdural hematoma	
	Cerebral vascular accident	

*AV*, arteriovenous.
